# P-1217. In Vitro Activity of Ledaborbactam (VNRX-5236) in Combination with Amoxicillin or Tebipenem Against Mycobacterium abscessus

**DOI:** 10.1093/ofid/ofaf695.1410

**Published:** 2026-01-11

**Authors:** Carolyn M Shoen, Cassandra Chatwin, David A Six, Michael H Cynamon

**Affiliations:** Veteran's Health Research Institute, Syracuse, NY; Venatorx Pharmaceuticals Inc., Malvern, Pennsylvania; Venatorx Pharmaceuticals, Inc., Malvern, Pennsylvania; Veteran’s Health Research Institute, Syracuse, New York

## Abstract

**Background:**

*Mycobacterium abscessus* (Mabs) causes respiratory tract infections in cystic fibrosis patients, systemic infections in immunocompromised patients, and soft tissue abscesses. Treatment is problematic due to the innate resistance of this organism to most drugs. Amoxicillin (AMX) and tebipenem (TBP), are ineffective due to the presence of a β-lactamase, BLA_Mab_. Ledaborbactam (LED), a broad-spectrum boronic acid β-lactamase inhibitor being developed as an orally bioavailable etzadroxil prodrug, inhibits this β-lactamase. The *in vitro* activities of LED in combination with AMX or TBP against Mabs were evaluated by microtiter broth dilution.

**Figure:**
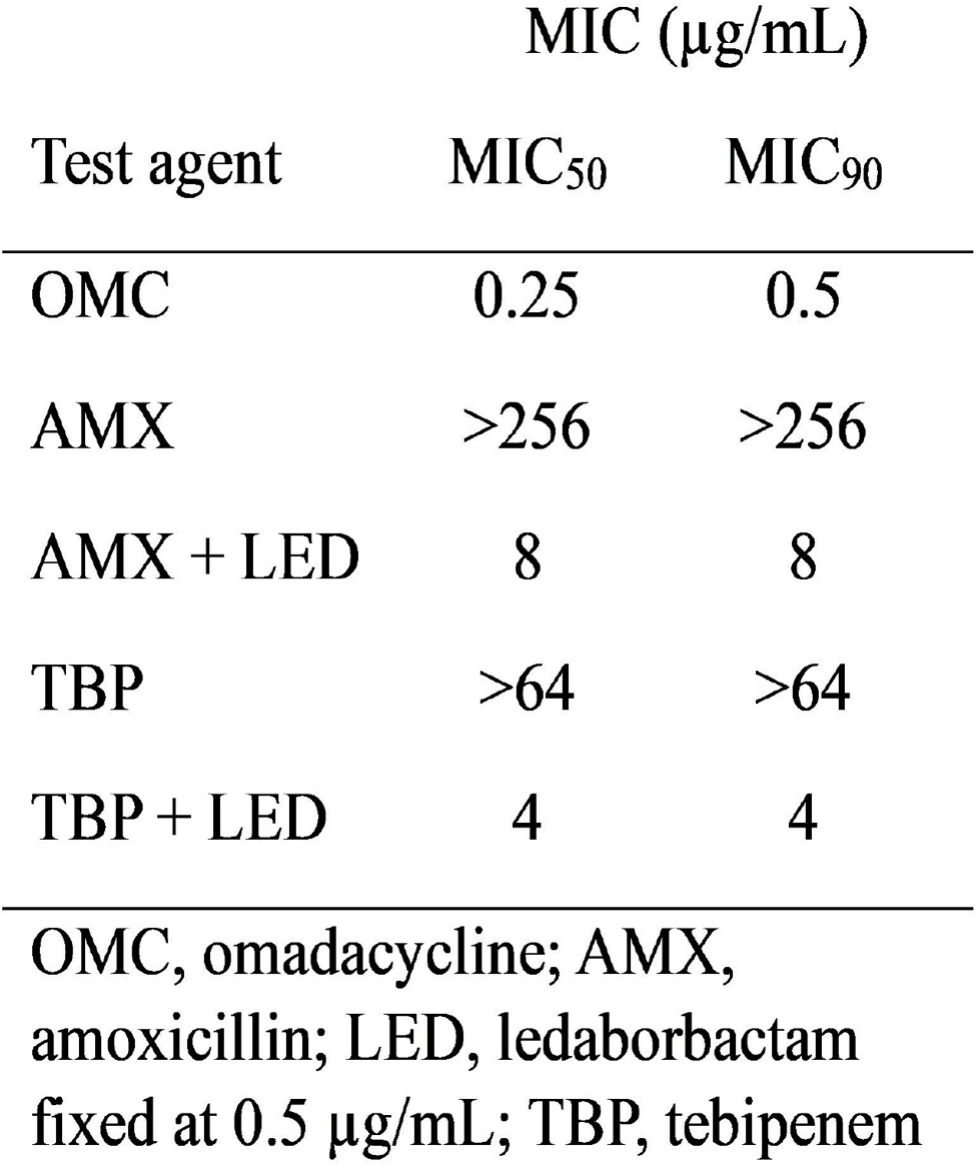
MIC50 and MIC90 of amoxicillin and tebipenem with and without the addition of ledaborbactam against 22 isolates of M. abscessus

**Methods:**

Clinical isolates were provided by Barbara Brown Elliott, Barbara Body, Jeffrey Starke, and CDC. ATCC 19977 was purchased from ATCC. AMX, TBP, LED, and the control drug, omadacycline (OMC) were dissolved in DMSO and frozen. MIC testing was accomplished using a modified CLSI method. Briefly, drugs were diluted in cation-adjusted Mueller Hinton broth (CAMHB). Polystyrene 96-well round-bottom plates were prepared with CAMHB and the drugs were added to the first well prior to being serially 2-fold diluted. Mabs isolates (21 clinical isolates and ATCC 19977) were diluted in CAMHB with or without 4 or 0.5 µg/mL of LED (final concentration of approximately 1.25 x 10^5^ CFU/mL). The cell suspension was added to each well. Plates were incubated at 37 °C in ambient air for 5 days prior to reading. The MIC assays were run in duplicate with QC strains run in parallel to confirm expected activities of all compounds. The MIC_50_ and MIC_90_ are defined as the concentration at which 50% and 90% of the 22 isolates were inhibited.

**Results:**

The MIC_50_ / MIC_90_ of AMX and TBP were >256 / >256 µg/mL, and 64 / >64 µg/mL respectively. LED markedly enhanced the activities of each of these agents. The MIC_50_ / MIC_90_ of AMX and TBP in combination with LED 0.5 µg/mL was 8 / 8 µg/mL and 4 / 4 µg/mL, respectively. LED at 4 µg/mL in combination with AMX or TBP yielded similar results.

**Conclusion:**

The addition of LED to both AMX and TBP dramatically improved the *in vitro* activities of these compounds against Mabs. Evaluating these combinations in animal models of Mabs infection will provide a better understanding of their potential to treat human disease.

**Disclosures:**

David A. Six, PhD, Venatorx Pharmaceuticals, Inc.: Employee Michael H. Cynamon, MD, Paratek: use of omadacycline for M. abscessus infections

